# Antibacterial phage ORFans of *Pseudomonas aeruginosa* phage LUZ24 reveal a novel MvaT inhibiting protein

**DOI:** 10.3389/fmicb.2015.01242

**Published:** 2015-11-06

**Authors:** Jeroen Wagemans, Anne-Sophie Delattre, Birgit Uytterhoeven, Jeroen De Smet, William Cenens, Abram Aertsen, Pieter-Jan Ceyssens, Rob Lavigne

**Affiliations:** ^1^Laboratory of Gene Technology, Department of Biosystems, KU LeuvenLeuven, Belgium; ^2^Laboratory of Food Microbiology, Department of Microbial and Molecular Systems, KU LeuvenLeuven, Belgium

**Keywords:** *Pseudomonas aeruginosa*, bacteriophages, LUZ24, phage–host interactions, MvaT

## Abstract

The functional elucidation of small unknown phage proteins (‘ORFans’) presents itself as one of the major challenges of bacteriophage molecular biology. In this work, we mined the *Pseudomonas aeruginosa*-infecting phage LUZ24 proteome for antibacterial and antibiofilm proteins against its host. Subsequently, their putative host target was identified. In one example, we observed an interaction between LUZ24 gp4 and the host transcriptional regulator MvaT. The polymerization of MvaT across AT-rich DNA strands permits gene silencing of foreign DNA, thereby limiting any potentially adverse effects of such DNA. Gel shift assays proved the inhibitory effect of LUZ24 gp4 on MvaT DNA binding activity. Therefore, we termed this gene product as Mip, the MvaT inhibiting protein. We hypothesize Mip prevents the AT-rich LUZ24 DNA from being physically blocked by MvaT oligomers right after its injection in the host cell, thereby allowing phage transcription and thus completion of the phage infection cycle.

## Introduction

To date, the gap between functional analysis of bacteriophage genes and the number of uncharacterized novel phage genome sequences is continuously increasing. The functional annotation of these so-called ‘ORFans’ is one of the greatest challenges of bacteriophage molecular biology. Previous studies suggest that bacteriophages target a wide variety of global processes in the host cell, many of them perhaps non-essential (Roucourt, 2009; Wagemans et al., 2014).

**FIGURE 1:**
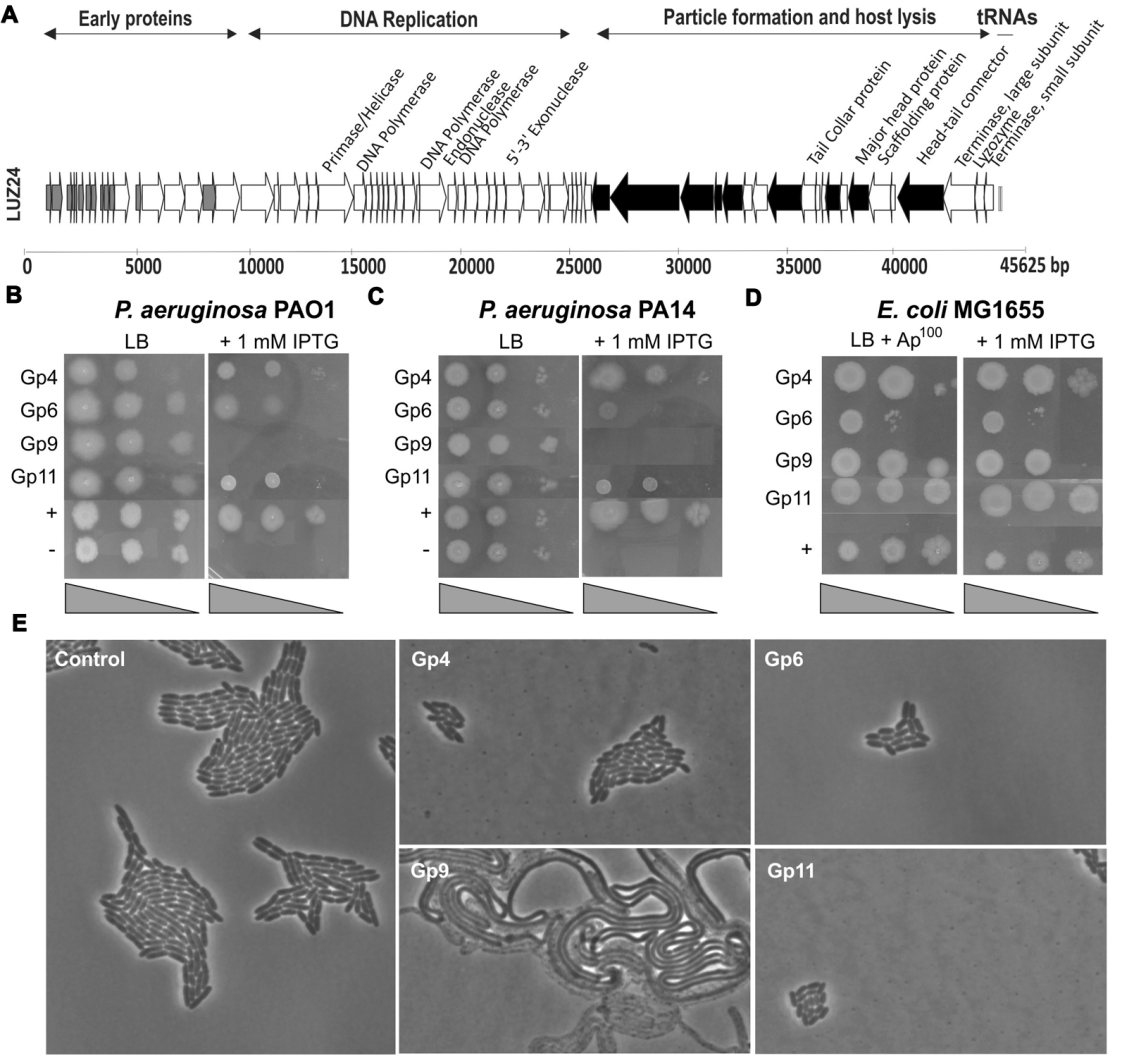
**(A)** LUZ24 genome annotation. The arrows represent the predicted open reading frames and point in the direction of transcription. Experimentally verified structural genes are indicated in black. Thirteen early LUZ24 proteins (their genes marked in gray) were evaluated for inhibition of bacterial growth. **(B–D)** Screening for growth-inhibitory LUZ24 early proteins by recombinant expression in *Pseudomonas aeruginosa* and *Escherichia coli*. Only the four proteins with a growth-inhibitory effect are shown. Serial dilutions of *P. aeruginosa* PAO1 **(B)** (10^0^, 10^-2^, 10^-4^), PA14 **(C)** (10^0^, 10^-2^, 10^-4^), and *E. coli* MG1655 **(D)** (10^0^, 10^-1^, 10^-2^) cells containing the different phage proteins were spotted on media with (right) and without (left) IPTG induction, together with a positive (*P. aeruginosa*::phiKMV gp5) and negative (empty vector construct) control. **(E)** *P. aeruginosa* PAO1 morphology after LUZ24 phage protein expression. Microscopic recording of *P. aeruginosa* PAO1::phage protein cells after growth for 5 h in the presence of IPTG.

LUZ24, a *Pseudomonas aeruginosa*-infecting podovirus, encapsulates a linear double stranded DNA molecule consisting of 45,625 bp and encoding 68 proteins (Ceyssens et al., 2008; **Figure 1A**). Promoter trap experiments revealed the presence of seven σ^70^-specific promoter regions, spread across the genome. This indicates a full dependency of LUZ24 on the transcriptional machinery of its host throughout the entire infection cycle. The early genome region almost only contains unique proteins lacking any similarities with proteins in the databases. One exception is the non-inhibitory gp2, which is conserved in PaP3 (Tan et al., 2007), PA11 (Kwan et al., 2006) and the entire *phiKMVlikevirus* genus (Ceyssens et al., 2006), suggesting a critical role in infection. The middle genome region encodes a DNA replication module, and the late genes, transcribed from the minus strand, are involved in particle formation and host lysis (Ceyssens et al., 2008).

Histone-like nucleoid structuring proteins (H-NS) exert a crucial role in the compaction of bacterial chromosomes and control of gene expression. Although widespread in Gram-negative bacteria, H-NS-like proteins usually exhibit low sequence homology (Dame et al., 2005). For example, MvaT of *P. aeruginosa* only shares 18% identical amino acids with the *Escherichia coli* H-NS (Tendeng et al., 2003). A second H-NS family member (MvaU), 51% identical and 68% similar to MvaT, appears to play a less prominent role in the control of gene expression in *P. aeruginosa* compared to MvaT (Vallet et al., 2004; Vallet-Gely et al., 2005).

Despite the lack of sequence conservation, all H-NS like proteins display an evolutionarily conserved structural and functional organization in two modules: an N-terminal oligomerization domain which adopts an α-helical structure (Bertin et al., 1999) and a more conserved C-terminal DNA binding domain (Bertin et al., 2001; **Figure 3**). Both domains are separated by a flexible linker region (Dorman et al., 1999).

A dense organization of the bacterial DNA by MvaT is established by the formation of bridges between adjacent DNA molecules, probably by dimerization of the oligomerization domain, leaving two exposed independent DNA binding domains (Dame et al., 2005). However, MvaT does not only form dimers to create bridges between DNA tracts. A central portion of the protein (amino acids 35–62), partly containing the N-terminal domain and the flexible linker, is involved in the formation of higher-order oligomers. These oligomers can easily bind across DNA, thus forming extensive nucleoprotein filaments and permitting gene silencing (Castang and Dove, 2010; Winardhi et al., 2012). MvaT has a distinct preference for binding AT-rich DNA regions, which facilitates the silencing of foreign DNA, thereby limiting any potentially adverse effects of inheriting such xenogeneic DNA (Navarre et al., 2007; Castang and Dove, 2010). Accordingly, MvaT is a global repressor of motility, resistance, and virulence genes that are often housed on AT-rich pathogenicity islands, acquired through horizontal gene transfer (Dorman and Kane, 2009; Ali et al., 2012).

Previous studies suggest that lytic bacteriophages target a wide variety of global processes in the host cell to establish a favorable environment for phage reproduction (Roucourt, 2009). One example is the 11 kDa protein gp5.5 of bacteriophage T7 which binds and inhibits H-NS of its host *E. coli* (Liu and Richardson, 1993). T7 phages lacking gene 5.5, which is one of the most highly expressed genes during T7 infection, remain viable but give reduced plaque size and burst yield in wild type *E. coli* strains (Studier, 1981). Gp5.5 specifically interacts with the central oligomerization domain of H-NS, thereby disrupting higher-order H-NS-DNA complexes and probably inhibiting H-NS gene silencing of the incoming foreign phage DNA (Ali et al., 2011; Zhu et al., 2012). Another example is coliphage T4 protein Arn which was also shown to interact with its host’s H-NS protein. Arn inhibits the DNA binding activity of H-NS by mimicking a DNA molecule and neutralizes the gene silencing effect of H-NS (Ho et al., 2014).

In this manuscript, the intracellular interactions between *P. aeruginosa* (66.6% GC) and the lytic podovirus LUZ24 (52.2% GC) which occur early in phage infection, were investigated. First, four novel antibacterial early LUZ24 proteins were identified. Further protein–protein interaction screens identified MvaT as the host target protein for one of these proteins, LUZ24 gp4. Gel shift assays using recombinant MvaT and LUZ24 gp4 proved an inhibitory effect of LUZ24 gp4 on MvaT DNA binding activity, potentially enabling this AT-rich phage to escape bacterial transcriptional silencing and proceed to complete its lytic infection cycle.

## Materials and Methods

### Expression in *Pseudomonas aeruginosa* and Bacterial Growth/Biofilm Formation Experiments

All phage genes were cloned in a Gateway entry vector using the pENTR/SD/D-TOPO cloning kit (Invitrogen; Primers used for PCR are listed in Supplementary Table S1). Subsequently, the genes were transferred to the *E. coli* – *P. aeruginosa* shuttle expression vector pUC18-mini-Tn7T-Lac (Choi et al., 2005), which was made Gateway compatible. Cotransformation of 250 ng of the pUC18-mini-Tn7T-Lac constructs and pTNS2 by electroporation to *P. aeruginosa* PAO1 or PA14 allowed single-copy integration of the phage proteins in the *Pseudomonas* genome (specifically in the Tn7 site between *PA5548* and *PA5549*) under the control of an IPTG-inducible lac promoter which was verified using PCR and DNA sequencing (Choi et al., 2005, 2006).

*Escherichia coli* and *P. aeruginosa* cells were grown at 37°C in Lysogeny Broth (LB) and on LB, artificial sputum medium (Sriramulu et al., 2005) or M9 minimal medium (Sambrook and Russell, 2001) plates, supplemented with 0.1 mg ml^-1^ ampicillin, 0.03 mg ml^-1^ gentamicin and/or 1 mM IPTG, if required. As negative and positive control in the expression experiments, *P. aeruginosa* cells containing an empty vector construct and inhibitory protein gp5 of phage phiKMV (unpublished data), respectively, were used. Growth curves were generated using a Bioscreen CTM spectrophotometer (Growth Curves USA).

Biofilm formation was assessed using the Calgary biofilm device (Ceri et al., 1999). Overnight cultures were diluted 200-fold in LB medium with or without IPTG in the 96 wells of a microtiter plate. Each plate contained four replicas per construct, arranged in a way to minimize spatial influence on biofilm formation, and three biological replicates were analyzed. Once the pegs were placed in the wells, the plate was sealed to reduce evaporation. Biofilms were then grown for 24 h at 37°C without shaking. The quantitative analysis of the biofilm formation and planktonic survival was performed as described previously (Cornelissen et al., 2011).

### Time-lapse Microscopy

A stationary culture of *P. aeruginosa* PAO1 mutant cells, containing a single-copy expression construct of the different phage proteins was diluted a thousand times and spotted on an LB agar pad containing 1 mM IPTG to induce expression. Subsequently, the growth of a single cell was recorded in real time for 5 h with a temperature controlled (Okolab Ottaviano) Ti-Eclipse inverted microscope (Nikon) equipped with a TI-CT-E motorized condensor and a CoolSnap HQ2 FireWire CCD-camera. Images were acquired using the NIS-Elements AR 3.2 software (Nikon) as described previously (Cenens et al., 2013).

### Yeast Two-hybrid Interaction Assay

Yeast two-hybrid analysis in *Saccharomyces cerevisiae* AH109 (James et al., 1996) was performed using a random genomic fragment *P. aeruginosa* PAO1 prey library (Roucourt et al., 2009). Bait proteins were tested for self-activation of the HIS3, ADE2, and MEL1 reporter constructs by transformation (Gietz and Woods, 2002) of the pGBT9 phage gene bait constructs together with an empty pGAD424 prey vector or the unrelated prey Gpa1p (the α-subunit of a G protein involved in pheromone signaling in yeast). Subsequently, the bait-containing AH109 cells were transformed with the prey library following the Gietz protocol (Gietz and Schiestl, 2007). Selection of positive colonies was done using synthetic defined minimal medium as described previously (Wagemans et al., 2014).

### Recombinant Production of MvaT and LUZ24 gp4

Recombinant LUZ24 gp4 with a C-terminal *Strep*-tag II (Strep, SAWSHPQFEK) was produced by cloning the corresponding gene in pEXP-5 using the pEXP-5-CT/TOPO TA cloning kit (Life Technologies). Recombinant MvaT, on the other hand, was produced in pCDF-1b (Merck Millipore) fused to a C-terminal hexahistidine tag. After verification by PCR clone analysis and DNA sequencing, *E. coli* BL21 (DE3) pLysS cells carrying the expression constructs were grown in two times 500 ml LB to an OD_600_ of 0.8 and subsequently induced with 1 mM IPTG for 3 h at 37°C.

Expression was ended by centrifugation and the cell pellet was lysed in 100 mM Tris pH7.6, 150 mM NaCl, 0.1 mg/ml lysozyme, 1 mM Pefablock (Merck), and 10 μg/ml DNase I. After incubation on ice for 30 min, the lysates were sonicated (10 cycles of 30 s) and centrifuged. Purification of the His-tagged proteins was performed on an Äkta Fast Protein Liquid Chromatograph (FPLC; GE Healthcare) using a 1 ml Protino Ni-NTA column (Macherey-Nagel). As there were still impurities in the protein sample, a second purification using Co^2+^-NTA was performed. Therefore, a self-made chromatography column (Bio-Rad) containing 1 ml Ni-NTA agarose beads (Qiagen) was first stripped using 1 ml 50 mM EDTA at a pH of 8.0 to remove all Ni^2+^ ions. Then, after washing the column with ultrapure water, 1 ml 100 mM CoSO_4_ (Sigma-Aldrich) was loaded on the beads to prepare a Co-NTA column, which was equilibrated with buffer (50 mM Tris pH 7.5, 150 mM NaCl) supplemented with 10 mM imidazole. Subsequently, the concentrated and dialyzed protein sample was loaded and washed three times in the same buffer using successively 10 mM (10 ml), 30 mM (5 ml), and 75 mM (5 ml) imidazole. The His-tagged protein was finally eluted in five times 1 ml buffer containing 300 mM imidazole, concentrated and dialyzed.

Purification of the Strep-tagged phage protein was done using a Poly-Prep Chromatography column (Bio-Rad) containing 1 ml equilibrated (100 mM Tris pH 8.0, 150 mM NaCl, 1 mM EDTA) *Strep*-Tactin^®^Sepharose beads (IBA, Göttingen, Germany). After loading the cleared lysates, the beads were washed with 10 column volumes washing buffer (100 mM Tris pH 8.0, 150 mM NaCl, 1 mM EDTA) and eluted with five times 1 ml *Strep*-tag elution buffer containing D-desthiobiotin (IBA). After concentration by ultrafiltration, the protein was dialyzed to 100 mM Tris pH 8.0, 150 mM NaCl, and 1 mM EDTA to remove D-desthiobiotin.

### Coprecipitation Assay

To verify the interactions *in vitro*, a pull down assay was performed. Therefore, 50 μl Ni-NTA agarose beads (Qiagen), equilibrated with washing buffer containing 50 mM Tris pH 8.0 and 150 mM NaCl, were used to immobilize the His-tagged bait protein. After 20 min incubation and two washing steps, a 10-fold excess of prey protein was added. The proteins and beads were again incubated for 20 min and washed twice. Finally, the Ni-NTA agarose beads were boiled at 95°C for 5 min to release the bound proteins. After centrifugation, the supernatant was analyzed using SDS-PAGE and Western blot.

### Gel Shift Assay

The MvaT sample (20 μM) was first incubated for 15 min at room temperature with increasing concentrations (0–40 μM) of phage protein in DNA binding buffer [20 mM 4-(2-hydroxyethyl)piperazine-1-ethanesulfonic acid (HEPES, Acros Organics) pH 7.6, 10 mM ammonium sulfate, 1 mM DTT, 3% v/v Tween 20 and 30 mM NaCl]. Bovine serum albumin (BSA, Thermo Scientific) served as a negative control. After addition of 200 ng DNA, the 10 μl samples were incubated for another 15 min and run at 4°C with DNA loading dye (Thermo Scientific) on a non-denaturing polyacrylamide gel [8% (v/v) 37.5:1 acrylamide/bisacrylamide, 0.025% (v/v) glycerol, 0.01% (v/v) APS and 0.001% (v/v) TEMED in TBE (89 mM Tris, 89 mM boric acid, and 2 mM EDTA)] in TBE running buffer. Finally, the gel was stained for 20 min in TBE supplemented with ethidium bromide and visualized using UV illumination.

As MvaT specifically binds to the *ptxS* upstream region of *P. aeruginosa* (Westfall et al., 2004), this 150 bp DNA fragment was amplified from *P. aeruginosa* PAO1 genomic DNA with primers 5′-GATTGATCGCTTTCTACCGAC-3′ and 5′-CATGAGGCCAGGACGTTGTTC-3′ and used as a positive control in the gel shift assays.

## Results

### Screening for Growth and Biofilm Inhibitory Early Phage Proteins

To unravel the function of unknown phage genes, the LUZ24 genome map was analyzed. Similar to previous work (Roucourt and Lavigne, 2009; Wagemans et al., 2014), we aimed to identify LUZ24-encoded inhibitory functions among those genes encoding small (less than 250 amino acids) cytoplasmic proteins, expressed early after infection. As such, 13 LUZ24 genes were selected, based on the clear genome organization (**Figure 1A**, marked in gray, listed in Supplementary Table S2) and were conditionally expressed in single copy in *P. aeruginosa* PAO1 to assay their growth inhibitory potential.

**FIGURE 2:**
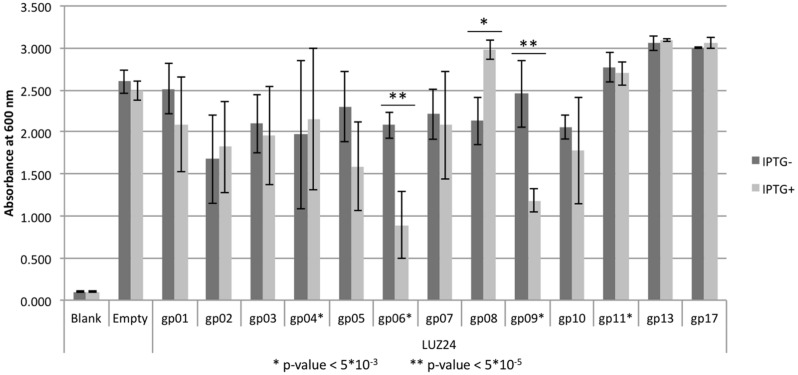
**Biofilm formation inhibition assay.** Biofilms were grown on cones with or without IPTG to induce expression of early phage protein. After 24 h the mean biofilm was scored by measuring optical density at 600 nm, represented on the y-axis. Three independent replicas were performed, starting from different overnight cultures with each four repeats per replica, resulting in a 12-fold repetition. Error bars represent the standard error, the asterisk (^∗^) and double asterisk (^∗∗^) mark significant values (two-tailed *t*-test) with respectively P < 5^∗^10^-3^ and < 5^∗^10^-5^.

Of the thirteen selected LUZ24 genes, only gp4, gp6, gp9, and gp11 impaired growth of *P. aeruginosa* PAO1 in terms of OD increase or colony formation in liquid or solid media (**Figure 1**). More specifically, whereas induced expression of gp4, gp6, and gp11 generally slowed down growth (**Figure 1E** and Supplementary Figure S1), expression of gp9 caused severe filamentous growth and prevented colony formation (**Figures 1B,E**). The biofilm forming capacity of *P. aeruginosa* PAO1 expressing early phage proteins was tested using a standard Calgaray device-based assay (Ceri et al., 1999). The antibacterial proteins gp6 and gp9 also result in reduced biofilm formation, while gp4, gp11 and the non-inhibitory proteins did not negatively impact biofilm formation (although an increase in biofilm formation was observed for LUZ24 gp8; **Figure 2**).

Next, we verified the growth-inhibitory effect of the phage proteins on PA14, a clinical *P. aeruginosa* isolate (Rahme et al., 1995; **Figure 1C**). While gp4 appears to have no inhibitory effect on *P. aeruginosa* PA14, gp6 has stronger inhibition compared to its PAO1 counterpart. Gp9 and gp11, on the other hand, cause a similar phenotype in both strains. Since gp9 was the only protein that completely inhibited proliferation of *P. aeruginosa* PAO1 and PA14, this *P. aeruginosa* PA14::LUZ24 gp9 strain was plated on LB lacking IPTG one and 3 h after induction with 1 mM IPTG (early in exponential phase at OD_600_ 0.1). The results (Supplementary Figure S2) show that gp9 is bactericidal for *P. aeruginosa* PA14, since 3 h after induction, the number of viable cells reduced with approximately one log unit.

Finally, the expression assay was performed in *E. coli* MG1655 (Blattner et al., 1997; **Figure 1D**). Episomal expression from the pUC18-mini-Tn7T-Lac construct did not result in an inhibitory effect on this *E. coli* strain, indicating the target is specific for *P. aeruginosa* or not essential for *E. coli* growth under these experimental conditions.

### *In silico* Analysis of Growth-inhibitory Phage Proteins

Based on BLASTp (Altschul et al., 1990; Gish and States, 1993) database searches, no function could previously be attributed to the different inhibitory proteins (Ceyssens et al., 2008). Further *in silico* homology analyses of the antibacterial LUZ24 proteins were performed using HMMER (Finn et al., 2011). All gene products are conserved (amino acid identity > 95%) among *Pseudomonas* infecting LUZ24-like phages PaP3, PaP4, and MR299-2. However, no significant similarities (e-value < 1 × 10^-5^) to any other known proteins were found. In addition, CDART (Geer et al., 2002), CDD (Marchler-Bauer et al., 2013) and Pfam 27.0 (Punta et al., 2012) searches revealed no conserved domains in any of the proteins. Finally, similar secondary structures were looked for using Phyre2 (Kelley and Sternberg, 2009) and HHPred (Soding et al., 2005), which provided no additional information. No secondary structures could be predicted. These results confirm the unique nature of all four inhibitory LUZ24 proteins.

### Exploratory Protein–Protein Interaction Analysis Using Yeast Two-hybrid (Y2H) and *In vitro* Pull Down Analysis

To explore the molecular background causing the inhibitory effects of the LUZ24 proteins, a Gal4p-based Y2H analysis in *S. cerevisiae* AH109 was performed using the inhibitory phage proteins as bait against a random genomic fragment library of *P. aeruginosa* PAO1 (Roucourt et al., 2009). The confirmed results are shown in **Table 1** and were reproducible in an independent Y2H experiment using fresh yeast cells. Moreover, the specificity of the interactions was examined using non-related bait and prey constructs. Finally, the α-galactosidase activity in milliunits per ml per cell was determined, which allows a relative comparison of the different interaction strengths (**Table 1**).

**Table 1:** Summary of phenotypic and protein–protein interaction analyses of the inhibitory LUZ24 proteins.

Protein	Growth in *Pseudomonas aeruginosa^∗^*		Growth in *Escherichia coli^∗^* MG1655	Prey identification by Y2H	Functional prediction of the Y2H target	α-Gal activity^$^
	PAO1	PA14				
Gp4	+/-	+	+	PA4315	MvaT, transcriptional regulator	23.07 ± 1.46
Gp6	+/-	+/-	+	/		
Gp9	-	-	+	PA0298 PA0259	SpuB, glutamylpolyamine synthetase Hypothetical protein	8.04 ± 0.36 Too weak
Gp11	+/-	+/-	+	N/A		

*^∗^Growth after induction: - no growth, +/- less growth, + normal growth, N/A not available. $ The α-galactosidase activity in milliunits per ml per cell. Because the weaker interaction between gp9 and PA0259 falls outside the sensitivity range of the α-galactosidase activity assay, no value could be calculated.*

**FIGURE 3:**
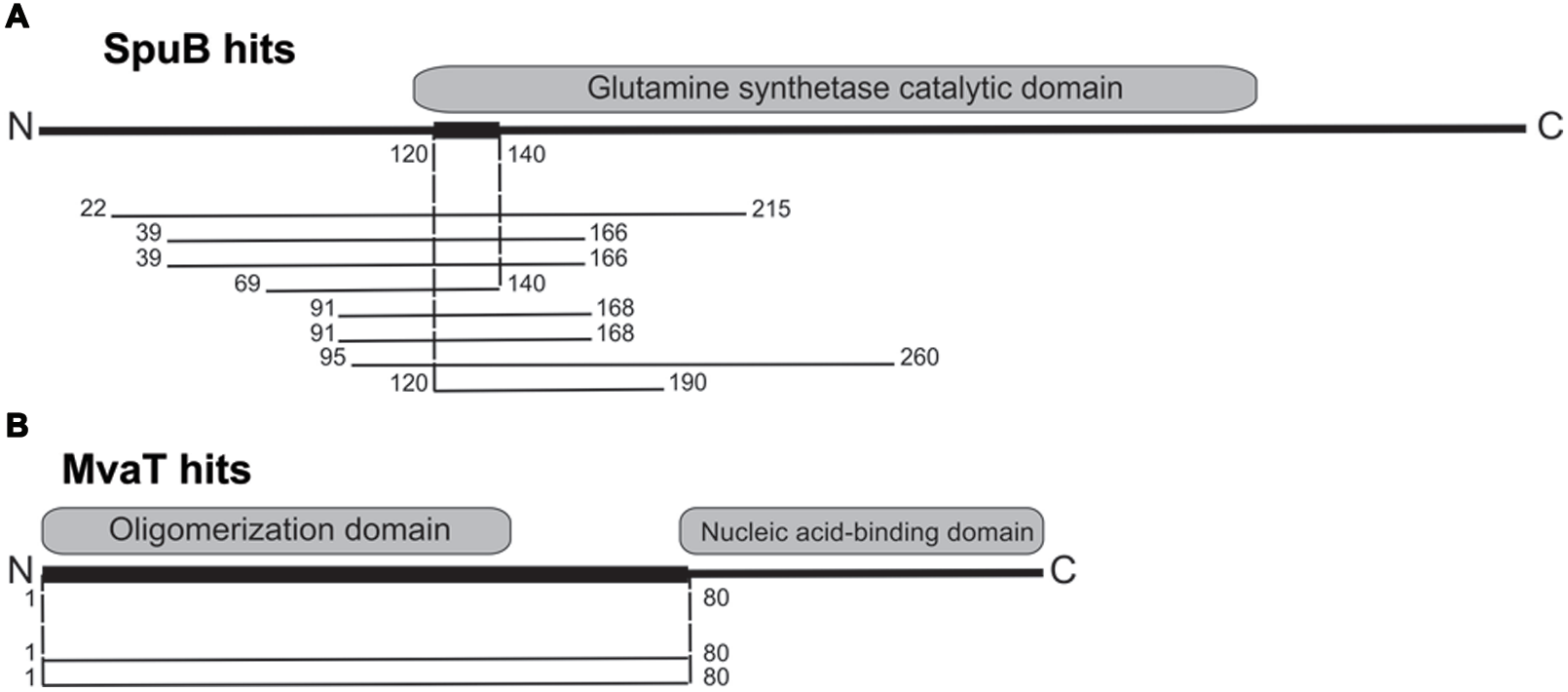
**Y2H results for LUZ24 gp9 and gp4. (A)** For LUZ24 gp9, eight positive colonies containing six different overlapping spuB fragments were identified. The narrow interaction domain spans amino acids 120–140, falling inside the catalytic domain of SpuB, which is in total 452 amino acids long. **(B)** LUZ24 gp4 potentially interacts with Pseudomonas protein PA4315 (MvaT). Since only two positive colonies harboring the same DNA fragments were found, the interaction domain can only be delineated to amino acid 1–80 out of this 124 amino acid-long MvaT protein. This encompasses the oligomerization domain.

Using LUZ24 gp4 as bait, one potential interaction partner in *P. aeruginosa* was revealed, the PA4315-encoded transcriptional regulator MvaT (Winsor et al., 2011). Although the *Pseudomonas* genome was represented multiple times in the used Y2H library, no overlapping PA4315 fragments could be found, so the interaction domain could only be delineated from amino acid 1 to 80 of this 124 amino acid protein, encompassing the oligomerization domain (**Figure 3**). With an α-galactosidase activity of 23.07 ± 1.46 milliunits (per ml substrate per yeast cell) the MvaT-LUZ24 gp4 interaction is the strongest identified interaction.

**FIGURE 4:**
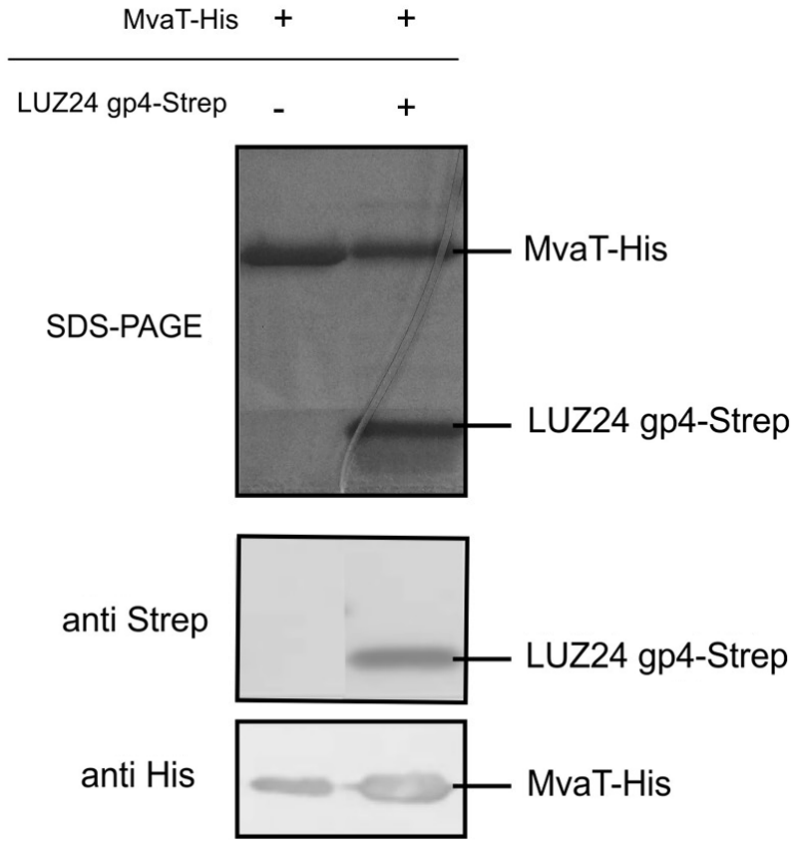
**MvaT pull down.** *In vitro* coprecipitation assay using MvaT-His bound on Ni-NTA beads as a prey and LUZ24 gp4 as a bait protein. The samples were resolved on a 16% Tris-tricine gel. Specific antibodies confirmed the presence of both proteins in Western blot.

To confirm the interaction, an *in vitro* pull down analysis was performed. First, recombinant His-tagged MvaT and recombinant Strep-tagged LUZ24 gp4 were produced in *E. coli* BL21 (DE3). These proteins were then used for *in vitro* coprecipitation assays using MvaT-His as a bait and the Strep-tagged phage protein as a prey. The results confirm the binding of both proteins *in vitro* (**Figure 4**). One can conclude the interaction between LUZ24 gp4 and MvaT is specific and reproducible.

For the bactericidal LUZ24 gp9, two potential interaction partners were identified, the first one being the γ-glutamylpolyamine synthetase SpuB, better known under the alternative name PauA2, which catalyzes one of the first steps of polyamine utilization in *P. aeruginosa* via the γ-glutamylation pathway (Yao et al., 2011). For this protein, the narrow interaction domain spans amino acid 120–140, which is located at the start of the catalytic domain (**Figure 3**). With an α-galactosidase activity of 8.04 ± 0.36 milliunits per ml per yeast cell this interaction forms an intermediately strong interaction. A second putative (weak) interaction partner for LUZ24 gp9 is the hypothetical protein PA0259. Both interactions were not confirmed using complementary techniques and were not explored further.

Since the MvaT-LUZ24 gp4 interaction was confirmed, we hypothesized that this early phage protein might inhibit MvaT-mediated gene silencing of the incoming foreign phage DNA immediately after infection of *P. aeruginosa*. With an average guanine and cytosine (GC-) content of 52.2% [determined using GC-Profile; (Gao and Zhang, 2006)] for phage LUZ24 (significantly lower compared to its host *P. aeruginosa* – 66.6%), it is indeed plausible that MvaT oligomers, which preferentially bind AT-rich DNA tracts, could mask the LUZ24 DNA and prevent it from being transcribed. As a defense mechanism, comparable to that of coliphage T7 gp5.5 (Liu and Richardson, 1993) or coliphage T4 Arn (Ho et al., 2014) on H-NS, the phage might produce a protein that binds and thereby inhibits MvaT.

**FIGURE 5:**
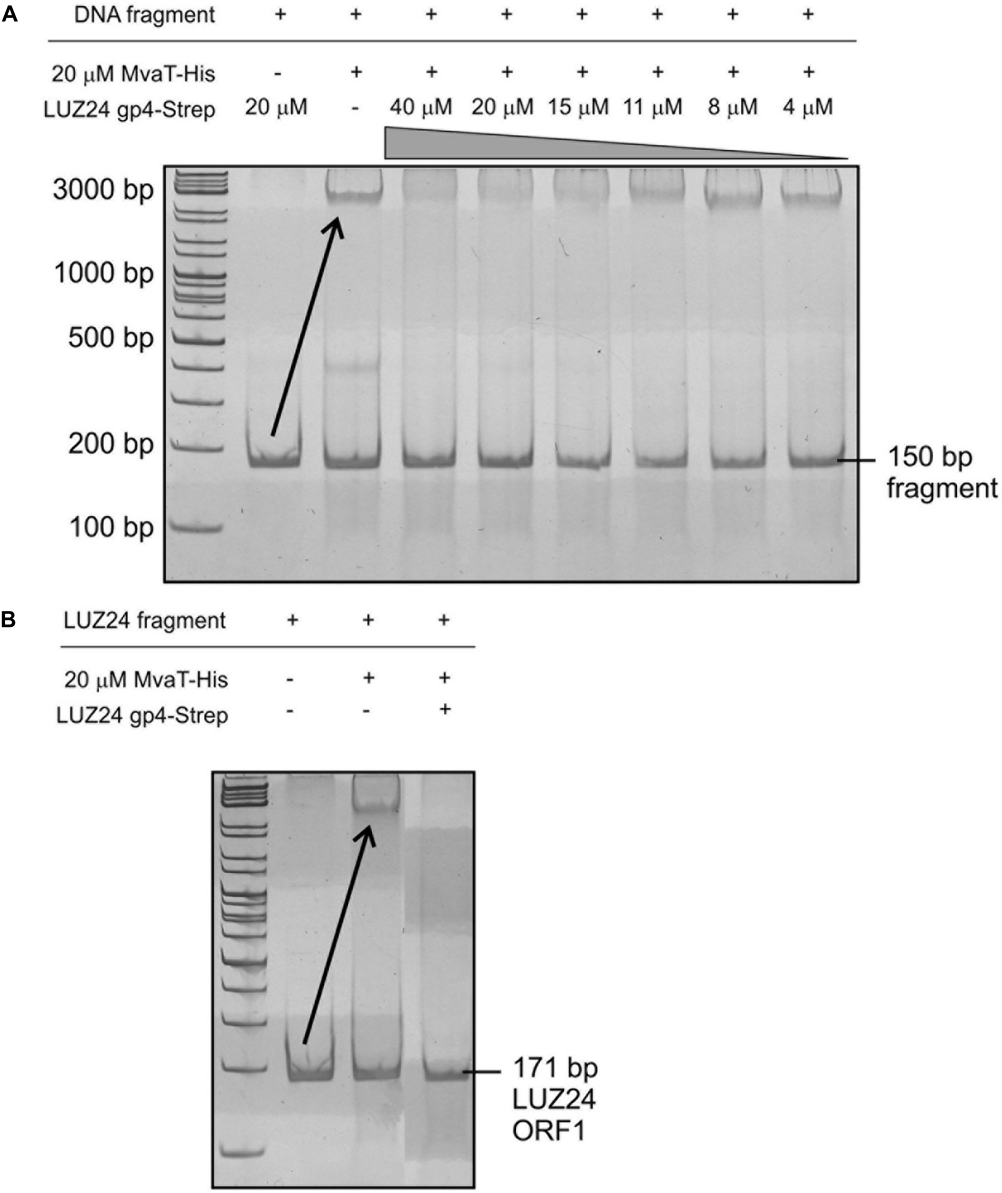
**MvaT gel shift assays. (A)** The MvaT protein specifically binds a 150 bp fragment in the *ptxS* upstream region as proven by a gel shift. LUZ24 gp4 alone, on the other hand, does not bind this DNA fragment. Adding increasing concentrations of the phage protein to MvaT, followed by incubation with the DNA fragment demonstrate the inhibitory effect of LUZ24 gp4 on MvaT DNA binding activity. **(B)** MvaT also binds LUZ24 genomic DNA (here only shown for ORF1), an effect that can be reversed by adding an equal amount of LUZ24 gp4.

To prove the hypothesis, recombinant MvaT-His and LUZ24 gp4-Strep were tested in a gel shift assay. Therefore, a *P. aeruginosa* 150 bp fragment, known to specifically bind MvaT (Westfall et al., 2004), was amplified and used as template. As shown in **Figure 5A** lanes 2 and 3, LUZ24 gp4-Strep does not bind the fragment, while MvaT-His does, as seen by the upward gel shift. By first incubating the same amount of MvaT-His together with different concentrations of LUZ24 gp4-Strep, MvaT is no longer able to bind the 150 bp fragment starting from approximately a 1:1 ratio of LUZ24 gp4-Strep:MvaT-His (**Figure 5A** lanes 4–9). Bovine serum albumin was used as a negative control (not shown in figure).

Next, the binding of MvaT to LUZ24 DNA was investigated using different LUZ24 early region PCR fragments as template in another gel shift assay (only shown for LUZ24 ORF1 in **Figure 5B**). These showed MvaT binds to all the tested fragments, which can be reversed by adding an equal amount of LUZ24 gp4 protein. To verify the specificity of this inhibition, recombinant His-tagged MvaU was produced in *E. coli* BL21 (DE3) and purified for a gel shift assay using the same template DNA as was used for MvaT. No inhibitory effect on the MvaU binding activity could be observed (data not shown).

## Discussion

In this work, we first selected thirteen unknown early phage ORFans of podovirus LUZ24 (Ceyssens et al., 2008). By expressing their encoded proteins individually in the host bacterium *P. aeruginosa*, we identified four antibacterial early phage proteins (gp4, gp6, gp9, and gp11). Although very unique, the early genome region encodes one protein, gp2, which is conserved in PaP3, PA11 and the entire phiKMV genus, suggesting a critical role in infection. This protein was shown to be non-inhibitory. Since we only tested for host growth and biofilm inhibition, additional research is needed to identify a putative function for this conserved protein. The technical limitation to produce modified lytic phages for LUZ24 presents a key hurdle toward their characterization, since gene product mutants/knock-outs could be a valuable tool toward functional elucidation of individual proteins within the phage.

We characterized the four antibacterial LUZ24 proteins using time-lapse microscopy and antibacterial assays in *P. aeruginosa* PA14 and *E. coli*. Since none of the inhibitory proteins showed any effect on *E. coli* MG1655, one may assume the identified antibacterial proteins are specifically tailored to influence the *P. aeruginosa* metabolism. To reveal the molecular background of their toxicity, we performed a protein–protein interaction analysis on the inhibitory LUZ24 proteins using a *P. aeruginosa* PAO1 yeast two-hybrid prey library (**Table 1**). While no host protein could be identified for gp6, the bactericidal gp9 putatively interacts with PA0298 and/or PA0259. However, these interactions could not yet be confirmed using complementary/independent approaches.

This confirmation was obtained for LUZ24 gp4. Our results show gp4 interacts with its host’s global transcriptional regulator MvaT and thereby inhibits the binding of the bacterial protein to the phage genome. Therefore, we termed this gene product as Mip, the MvaT inhibiting protein. Since MvaT has a distinct preference for binding AT-rich DNA regions, which facilitates the silencing of foreign DNA and limiting any potentially adverse effects of such xenogeneic DNA (Navarre et al., 2007; Castang and Dove, 2010), one can imagine Mip prevents the AT-rich LUZ24 DNA from being physically blocked by MvaT oligomers right after its injection in the host cell. The *mip* gene is one of the first LUZ24 genes transcribed after genome injection behind a strong σ^70^-promoter (Ceyssens et al., 2008), so it is plausible that sufficient amounts of Mip are produced even before host MvaT is able to find and bind the AT-rich LUZ24 DNA. Intriguingly, Mip does not inhibit the MvaT analog MvaU, which is 51% identical to MvaT. It is possible that MvaU plays a less prominent role in gene silencing of the incoming LUZ24 DNA than MvaT, as was observed previously for other gene regulation (Vallet et al., 2004; Vallet-Gely et al., 2005).

The MvaT inhibition strategy gives the phage a clear advantage, since a physical blockage of its DNA right after injection will compromise further steps in the infection cycle. Inhibition of MvaT by a phage-encoded protein will free the phage DNA, thereby allowing phage transcription and thus completion of the phage infection cycle.

Mip is a paralog of the previously characterized gp5.5 and protein Arn of coliphages T7 and T4, respectively. Sequence similarity searches revealed that gp5.5 homologs are found exclusively in the subset of *Autographivirinae*, short-tailed dsDNA phages that encode their own RNA polymerase, that infect bacteria of the family *Enterobacteriaceae*, but not in phages infecting *Vibrio* or *Pseudomonas* (Ali et al., 2011). This may well correlate to the fact that the H-NS like proteins of these other species bear almost no sequence similarity to *E. coli* H-NS (Nye and Taylor, 2003; Castang et al., 2008). Our results show that H-NS inhibiting proteins are more widespread than previously appreciated, and might be indispensable proteins to counter bacterial defense systems.

## Conflict of Interest Statement

The authors declare that the research was conducted in the absence of any commercial or financial relationships that could be construed as a potential conflict of interest.
