# Supplementary material for: Antibacterial phage ORFans of Pseudomonas aeruginosa phage LUZ24 reveal a novel MvaT inhibiting protein
Source: Front Microbiol. 2015 Nov 6;6:1242. doi: 10.3389/fmicb.2015.01242 (PMC4635203; doi:10.3389/fmicb.2015.01242)
Supplement: Supplementary file 1 [file Data_Sheet_1.DOCX]

# Supplementary Material

Table S1: Primers used for cloning of LUZ24 genes

| Gene | Forward primer | Reverse primer | Length in bp |
| --- | --- | --- | --- |
| *ORF1* | CACCATGTATCTTCTAGAGGGAC | TCAGGAGTTAATGAGAATG | 171 |
| *ORF2* | CACCATGAAAACTGTACTCACC | TCAGGCCGCCTCGCCGTTG | 555 |
| *ORF3* | CACCATGGATGAACTAACCGAGGA | TCATTTGGTCCACCCGCA | 219 |
| *ORF4* | CACCATGAAGTCCCCCTACGA | TCATTTCTTCAACCTCTTAATC | 141 |
| *ORF5* | CACCATGACTATCGCTATCGTG | TCAGTTGATACCCATCTCT | 84 |
| *ORF6* | CACCGTGAAACGCAATGACTATCGT | TCACCGCGCCCTGTTC | 183 |
| *ORF7* | CACCATGGATATCAAGGTGTGG | TTACACCTCGTAGCAATTC | 234 |
| *ORF8* | CACCATGAAACTCCAATACAACATGATTACC | TCAGTCCGGGAGGCCGA | 276 |
| *ORF9* | CACCATGAAATCCACTGAACC | TCAGTGGACCCGATGA | 147 |
| *ORF10* | CACCATGACTACTTTCACCATAAC | TTATTCCTCATACGGACC | 252 |
| *ORF11* | CACCATGAGGAATAAACCAATGATCGGCCA | TCACCAGTCCCTCGCGCA | 282 |
| *ORF13* | CACCATGCTTTCCCCCGCTAACAC | CTAGTTACCGTGGCCACGA | 153 |
| *ORF17* | CACCATGAATGACCTGAATAATC | CTATCTGTGTGCCAATAAC | 570 |

All PCRs were performed using Pfu polymerase following the standard guidelines (annealing temperature of 55 °C and 30 cycles)

Table S2: Selection of LUZ24 proteins

| Protein | Protein accession number | Size in amino acids |
| --- | --- | --- |
| Gp1 | YP_001671874 | 56 |
| Gp2 | YP_001671875 | 184 |
| Gp3 | YP_001671876 | 72 |
| Gp4 | YP_001671877 | 46 |
| Gp5 | YP_001671878 | 27 |
| Gp6 | YP_001671879 | 60 |
| Gp7 | YP_001671880 | 77 |
| Gp8 | YP_001671881 | 91 |
| Gp9 | YP_001671882 | 48 |
| Gp10 | YP_001671883 | 83 |
| Gp11 | YP_001671884 | 93 |
| Gp13 | YP_001671886 | 50 |
| Gp17 | YP_001671890 | 189 |


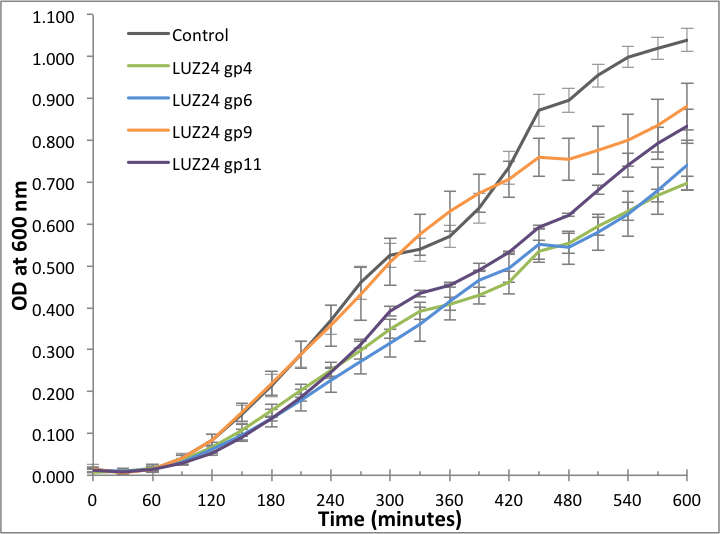


Figure S1: *P. aeruginosa* PAO1 growth kinetics after induction of LUZ24 phage protein expression. Growth was monitored over time by measuring the optical density (OD) of the bacterial culture at 600 nm. Phage protein expression was induced at the start of the experiment. Each data point represents the average of three independent measurements together with its standard deviation.


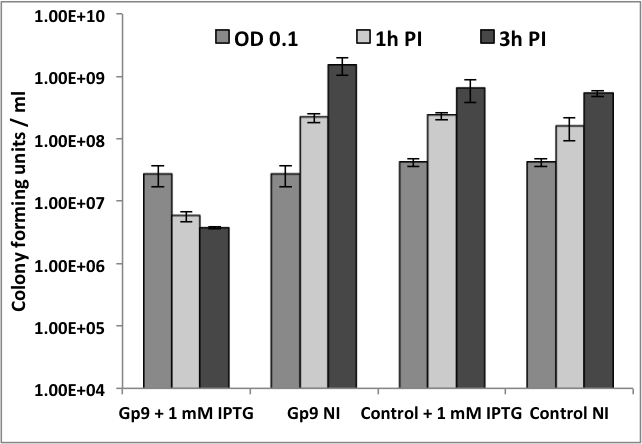


**Figure S2: Expression of LUZ24 gp9 in *P. aeruginosa* PA14.** In early exponential phase (OD 0.1), expression of LUZ24 gp9 was induced in a liquid LB culture. Subsequently, one and three hours post induction (PI) cells were plated on LB lacking IPTG. The number of viable cells in the sample was counted and plotted on a graph. As control the wild type strain containing an empty expression cassette and the non-induced (NI) samples were used, the latter showing a similar growth rate as the control strain. This experiment was performed for three independent samples, with the standard deviations shown on the graph.
